# Puma and Trail/Dr5 Pathways Control Radiation-Induced Apoptosis in Distinct Populations of Testicular Progenitors

**DOI:** 10.1371/journal.pone.0012134

**Published:** 2010-08-12

**Authors:** Mathieu Coureuil, Nicolas Ugolin, Marie Tavernier, Sylvie Chevillard, Vilma Barroca, Pierre Fouchet, Isabelle Allemand

**Affiliations:** 1 Laboratory of Gametogenesis, Apoptosis and Genotoxicity, INSERM, U967 Institute of Cellular and Molecular Radiobiology, CEA, Univ Paris VII, Fontenay aux Roses, France; 2 Laboratory of Experimental Cancerology, Institute of Cellular and Molecular Radiobiology, CEA, Fontenay aux Roses, France; INMI, Italy

## Abstract

Spermatogonia- stem cells and progenitors of adult spermatogenesis- are killed through a p53-regulated apoptotic process after γ-irradiation but the death effectors are still poorly characterized. Our data demonstrate that both intrinsic and extrinsic apoptotic pathways are involved, and especially that spermatogonia can be split into two main populations, according to apoptotic effectors. Following irradiation both *Dr5* and *Puma* genes are upregulated in the α_6_-integrin-positive Side Population (SP) fraction, which is highly enriched in spermatogonia. Flow cytometric analysis confirms an increased number of Dr5-expressing SP cells, and Puma-β isoform accumulates in α_6_-integrin positive cellular extracts, enriched in spermatogonia. *Trail−/−* or *Puma−/−* spermatogonia display a reduced sensitivity to radiation-induced apoptosis. The TUNEL kinetics strongly suggest that the extrinsic and intrinsic pathways, *via* Trail/Dr5 and Puma respectively, could be engaged in distinct subpopulations of spermatogonia. Indeed flow cytometric studies show that Dr5 receptor is constitutively present on more than half of the undifferentiated progenitors (Kit^−^ α_6_^+^ SP) and half of the differentiated ones (Kit^+^ α_6_^+^ SP). In addition after irradiation, Puma is not detected in the Dr5-positive cellular fraction isolated by immunomagnetic purification, while Puma is present in the Dr5-negative cell extracts. In conclusion, adult testicular progenitors are divided into distinct sub-populations by apoptotic effectors, independently of progenitor types (immature Kit-negative versus mature Kit-positive), underscoring differential radiosensitivities characterizing the stem cell/progenitors compartment.

## Introduction

Among the consequences of genotoxic stress, subfertility and transient sterility are an important issue for adult males. Injured germ cells, like in somatic self-renewing tissues, are located in the progenitor population, composed of mitotic spermatogonia that are the pre-meiotic cells in spermatogenesis. DNA damage results in apoptosis of part of the spermatogonia but resistant testicular stem cells allow afterwards the recovery of functional differentiation. As for somatic cells, apoptosis of damaged spermatogonia is controlled by the tumor suppressor p53, but its downstream apoptotic effector(s) remain far less characterized [1], [2].

Among the apoptotic factors, procaspases −2, −7, −8 and −9 are constitutively expressed in adult mouse spermatogonia [3]. After a genotoxic stress, the *Fas/CD95/Tnfrsf6* death receptor gene had been identified as a p53 target in somatic cells [4] and the involvement of the extrinsic death receptor pathway has been further evoked in germ cells. Nevertheless, the requirement for Fas/Fas-Ligand in radiation-induced apoptosis of testicular germ cells remains controversial [5], [6]. Trail/Dr5 pathway could represent a better candidate. In the mouse Dr5/Trail-R2/Tnfrsf10b is the only receptor of the ligand Trail (TNF-related apoptosis inducing ligand) and activation of this signaling pathway can trigger apoptosis of infectious and cancer cells [7]. *Dr5* is a p53-inducible gene, and *Dr5−/−* mice are viable but present impaired apoptotic response to irradiation [8], [9]. Trail induces *in vitro* apoptosis of normal testicular cells, *via* expression of Dr5 on spermatocytes [10], but the involvement of Trail/Dr5 pathway in stress-induced death of spermatogonia has not been assayed yet.

The role of the Bcl2 family, and therefore of the intrinsic/mitochondrial death pathway, in the control of germ cell development is known. The pro-apoptotic Bax and the anti-apoptotic Bcl-x_L_ are necessary as well as pro-apoptotic BH3-only proteins [11]–[13]. Nevertheless, the role of Bcl2 proteins in radiation-induced apoptosis of adult male germ cell is far less demonstrated, while some are known to be widely involved in genotoxic damage tissular response (i.e, Bax, Puma).

One reason may be the difficulty to access spermatogonia. Testicular stem cells and progenitors represent less than 10% of the adult germ cells and are located along the basal membrane of the seminiferous tubule, which also includes meiotic and haploid cells. According to histological criteria, undifferentiated spermatogonia include stem cells (A_single_) and less committed progenitors (A_paired_ and A_aligned_), whereas spermatogonia from A_1_ to B constitute the more differentiated sub-populations [14]. Immature spermatogonia can be identified on tissue sections by their expression of stem cell markers, like Plzf/Zbtb16 [15]. The improvement of their characterization allows their isolation by association of several stem cell markers. Thus, a α_6_-integrin-positive (α_6_^+^) population enriched in spermatogonia can be isolated after immunomagnetic purification [16]. Testicular germ cells display the Side Population (SP) phenotype - based on the Hoechst 33342 (Ho42) efflux -that characterizes stem cells [17], [18]. In combination with anti α_6_^+^-integrin pre-purification, SP criterion selects a fraction (α_6_^+^ SP) highly enriched in testicular stem cells. An additional screening of α_6_^+^ SP cells based on the expression of the c-Kit receptor allows the separation between immature (c-Kit negative) and differentiated spermatogonia (c-Kit positive) [19]–[21].

In order to identify the effectors responsible for genotoxic-induced apoptosis of spermatogonia, we demonstrate that different p53-regulated pathways are engaged: mitochondrial *via* Puma and extrinsic *via* Trail/Dr5. According to Dr5 expression, our results show that spermatogonia can be constitutively divided up into sub-populations that overlaps the traditional distribution -undifferentiated Kit^−^ *versus* differentiated Kit^+^- and potently reflects different death- sensitivities.

## Results

### γ rays induce Dr5 expression in spermatogonia in a p53-dependent manner

**Figure 1:**
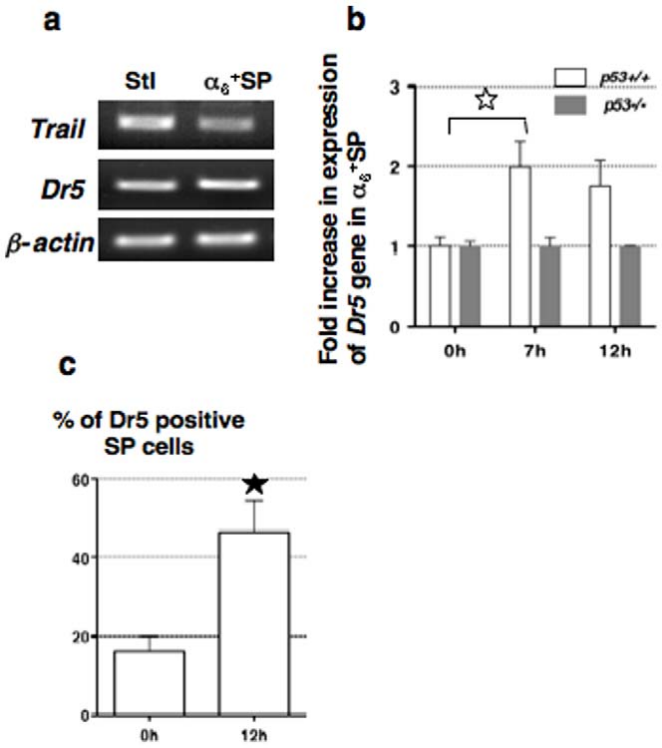
Dr5 receptor expression is induced in a p53-dependent manner on irradiated spermatogonia. (**a**) *Trail* and *Dr5* genes are expressed in Sertoli cells (Stl) and in α_6_^+^ SP cells, (negative for CD45 and highly enriched in spermatogonia); PCR amplifications (35 cycles) were carried out using 5 µL of first strand reaction for *Trail* and *Dr5* genes and normalized to *β-actin* PCR product (30 cycles). (**b**) Comparison of *Dr5* expression in *WT* or *p53−/−* sorted CD45^−^ α_6_^+^SP cells (highly enriched in spermatogonia), by semi-quantitative RT-PCR prior to, 7 and 12 hours after irradiation. Each expression level of *Dr5* was normalized to its value in non-irradiated *WT* cells and is indicated as a fold increase (± SEM, three independent experiments using pooled populations that were independently purified from 5 *WT* and 3 *p53−/−* males). A significant difference (star, p = 0.0232) was observed in *WT* cells prior to and 7 h post-IR. (**c**) *Wild-type* SP cells (n = 5) were analyzed independently for Dr5 receptor expression by FACS prior to and 12 h after irradiation, according to their DNA content. Mean percentages of Dr5- positive SP cells are shown (± SEM) and the star indicates a significant variation (p = 0.01033). See Figure S2 for details on flow cytometric analysis.

As the p53-controlled *Dr5* gene is involved in radiation-induced apoptosis of various somatic cells, we asked whether the Trail/Dr5 signaling pathway could be responsible for death of spermatogonia (Death marker evolution is presented in Figure S1). *Trail* and *Dr5* genes were expressed in primary Sertoli cells and in the testicular α_6_^+^ SP fraction (Fig. 1a). Then we measured the variation in *Dr5* gene expression levels in α_6_^+^ SP cells prior to and post-IR (post irradiation) (Fig. 1b): *Dr5* expression was two-fold higher than in non-irradiated cells and remained stable at 12 h. Consistent with these results, the number of SP cells expressing the Dr5 receptor was increased a 2.8 fold, 12 h post-IR (Fig. 1c and analysis details in Figure S2). Conversely, in irradiated *p53*−/− cells, *Dr5* expression level did not vary, demonstrating that the stress- induced upregulation of *Dr5* in damaged spermatogonia was p53-dependent.

### The Trail/Dr5 signaling cascade is the only Death Receptor pathway involved in radiation-induced apoptosis of spermatogonia

**Figure 2:**
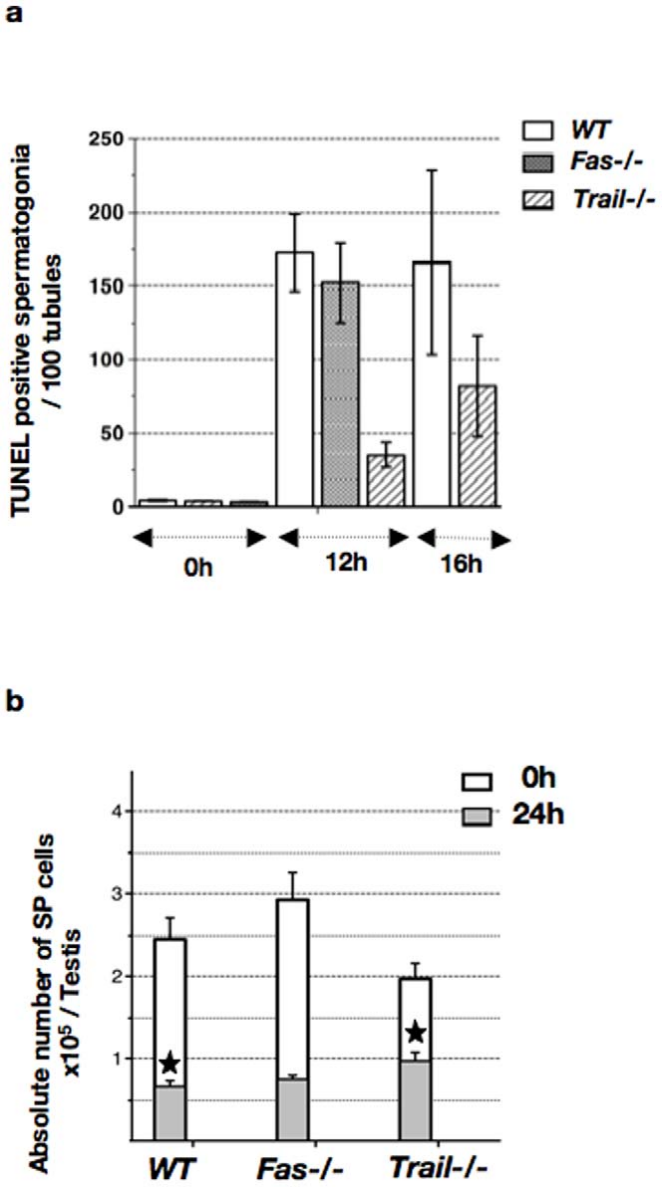
Trail/Dr5 is involved in irradiated spermatogonia apoptosis. (**a**) Comparison of TUNEL labeling between irradiated *Trail−/−*, *Fas−/−* and *WT* testes. The number of TUNEL-positive cells, located along the basal membrane, was determined prior to and post-IR, and indicated as the mean per 100 tubules (± SEM). Experiments were repeated 3 times and at least 200 tubules were counted in 2 males per genotype. (**b**) Viability of *Trail−/−*, *Fas−/−* and *WT* SP cells prior to and 24 h post-IR. Means of absolute number of SP cells per testis (± SEM) were determined after Ho42 and PI staining of testicular cell suspensions, analyzed in triplicates by FACS in the presence of fluorescent beads as an internal reference. The irradiated *WT* SP fraction was significantly lower than control (white star p<0.0001, n≥5). Irradiated *Trail−/−* SP significantly differs from *WT* SP (black star, p = 0.022, n≥5). Basal and irradiated *Fas−/−* and *WT* SP fractions did not differ (p≫0.05, n>5).

We further investigated the *in vivo* involvement of the Trail/Dr5 pathway by monitoring the death response of irradiated *Trail−/−* cells. Twelve hours post-IR, the number of TUNEL-positive *Trail−/−* spermatogonia was four-fold lower than that of *WT* cells (Fig. 2a). The number of positive *Trail−/−* cells was doubled at 16 h, while the number of TUNEL-positive *WT* spermatogonia remained constant. By contrast, the number of positive spermatogonia in irradiated *Fas−/−* males was comparable to that of *WT* counterparts. The lower sensitivity to radiation-induced apoptosis of *Trail−/−* germ cells was confirmed by *in situ* labeling for cleaved caspase-7 (data not shown).

Moreover, by monitoring at 24 h post-IR the absolute number of SP cells (Fig. 2b), we detected a 2.7-fold reduction in the viability of *WT* SP cells, while viability of *Trail−/−* SP cells was only a 2-fold decreased. In other words, the irradiated *Trail−/−* SP fraction was significantly higher than that of irradiated *WT* cells (1.4 fold), while there was no significant difference in the number of non-irradiated *WT* and *Trail−/−* SP cells. By contrast, viability of *Fas−/−* and *WT* SP cells was comparable.

In conclusion, these data demonstrate that the Trail/Dr5 extrinsic pathway is involved in the radiation-induced death of spermatogonia.

### γ rays induce Puma expression in spermatogonia in a p53-dependent manner

**Figure 3:**
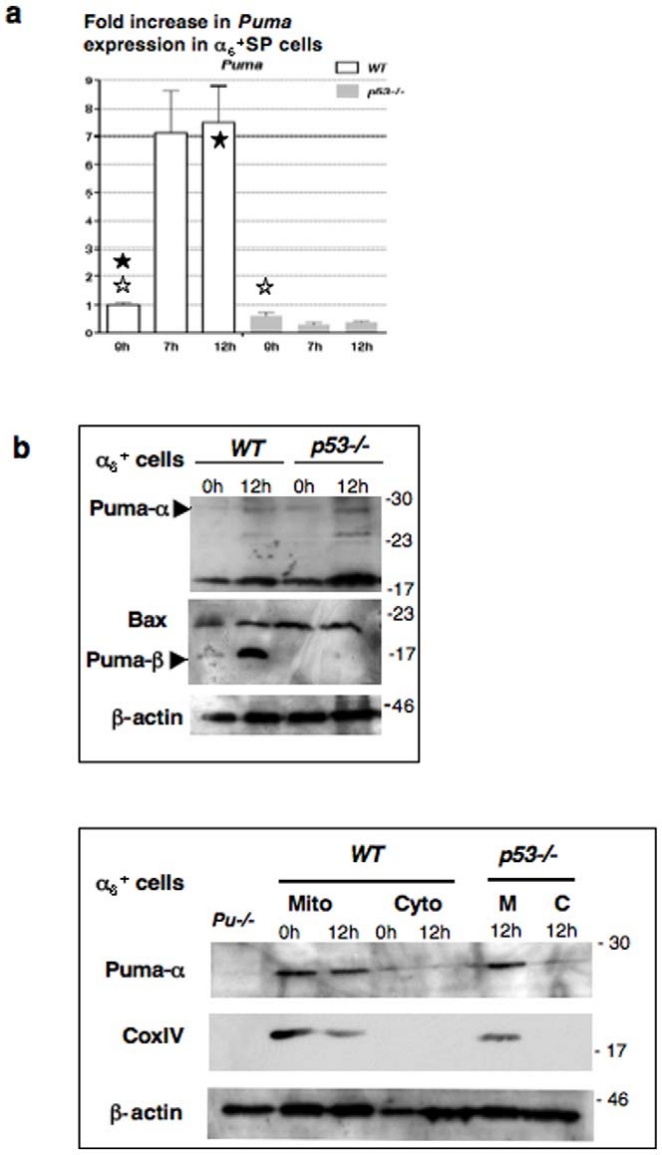
Increased *Puma* gene expression in a p53-dependent manner in α_6_^+^SP cells. (**a**) Comparison in *WT* or *p53−/−* sorted CD45^−^α_6_^+^SP cells (highly enriched in spermatogonia) by semi-quantitative RT-PCR prior to, 7 and 12 hours after irradiation. Each expression level was normalized to its value in non-irradiated *WT* cells and is indicated as a fold increase (± SEM, three separate experiments from pooled CD45^−^ α_6_^+^SP populations, which were independently purified from 5 *WT* and 3 *p53−/−* mice). A significant difference in *Puma* expression level was observed in *WT* α_6_^+^SP cells before irradiation and 12 h post-IR (black star, p = 0.015), and the basal level was significantly reduced in *p53−/−* α_6_^+^SP cells (white star, p = 0.00074). See Figure S3 for complete semi-quantitative RT-PCR analysis. (**b**) Puma expression in α_6_^+^ cells (testicular cell fraction enriched in spermatogonia after immunomagnetic purification) was determined by western-blot analysis. The α isoform was identified on the upper picture with the 9643 antibody, using *Puma−/−* cell lysate as a negative control and according to Callus and *col*. [41]. The Puma-β isoform was detected with the 4976 antibody and β actin was used as loading control. On the last picture, testicular α_6_^+^ cells were collected prior and 12 h post-IR, and separated into cytosolic and mitochondrial-enriched fractions for puma-α location and expression level; the sample purity was assayed using an anti-Cox IV antibody (mitochondrial marker) and β actin as loading control. Data presented are representative of 2 independent experiments and each α_6_^+^ population was purified from 3 males.

The previous data suggest that *Trail* inactivation did not protect all spermatogonia from radiation-induced apoptosis, which is mainly triggered by the Bcl2 family in somatic cells, through the intrinsic pathway. The pro-apoptotic members (e.g. Bax) interact with the mitochondrial membrane inducing cytochrome c release and consecutive pro-caspase-9 activation. Upstream, the subgroup of BH3-only proteins controls activities of pro- and anti-apoptotic Bcl2 family members. In order to identify Bcl2-related factors involved in spermatogonia death following irradiation, we analyzed several candidate genes by Real-Time PCR in α_6_^+^ SP cells. *Puma* (p53 upregulated modulator of apoptosis) was the only “apoptotic” gene exhibiting a significant 7- fold increase in irradiated α_6_^+^ SP cells (Fig. 3a and Figure S3) [22], [23]. This upregulation was directly correlated to the *p53* status as indicated by the absence of changes in RNA level in *p53−/−* α_6_^+^ SP cells.

Since *Puma* gene codes for different alternative transcripts we wondered which isoforms were expressed in healthy and damaged cells. Puma-α was detected by western-blot in α_6_^+^ populations enriched in spermatogonia (Fig. 3b upper). Its expression level appeared constant whatever the p53 status as confirmed in the mitochondrial fractions of the α6+ cells (Fig. 3b lower). Conversely, Puma-β accumulated in irradiated α_6_^+^ *WT* cells, while it remained undetectable in *p53−/−* α_6_^+^ populations (Fig. 3b upper). In parallel, Bax amounts remained constant whatever the conditions.

### Puma is involved in radiation-induced apoptosis of spermatogonia

**Figure 4:**
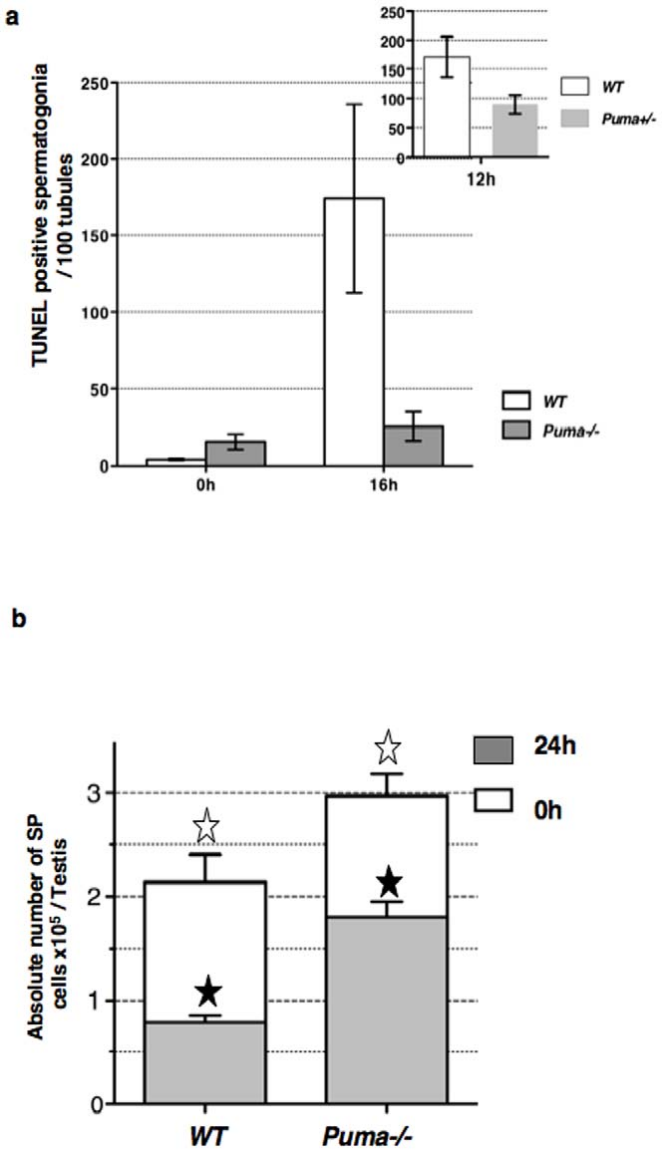
Puma is involved in spermatogonia radiation-induced apoptosis. (a) Comparison of TUNEL labeling between irradiated *Puma−/−* and *WT* testes. The number of TUNEL-positive cells, located along the basal membrane, was determined prior to and post-IR, and indicated as the mean per 100 tubules (± SEM). Experiments were repeated twice and at least 200 tubules were counted in 2 males per genotype. (**b**) Viability of *Puma−/−* and *WT* SP cells prior to and 24 h post-IR. Means of absolute number of SP cells per testis (± SEM) were determined after Ho42 and PI staining of testicular cell suspensions, which were analyzed by FACS in triplicates in the presence of fluorescent beads as an internal reference. The *WT* SP fraction significantly differs from the *Puma−/−* SP population prior (white star, p = 0.022; n = 5) and after irradiation (n = 5, black star p<0.0001).

To confirm the involvement of the BH3-only Puma in apoptotic radiosensitivity, dead spermatogonia of irradiated *Puma−/−* mice were counted (Fig. 4a). The number of TUNEL-positive *Puma−/−* spermatogonia was 7-fold lower than that of *WT* cells at 16 h. The pro-apoptotic effect of *Puma* was correlated to allele number as shown by the 2-fold reduction in TUNEL-positive *Puma+/−* spermatogonia at 12 h post-IR (insert Fig. 4a). In addition 24 h post-IR (Fig. 4b), viability of *Puma−/−* SP cells was only 1.6 fold decreased in comparison to the 2.7 fold reduction observed for *WT* SP cells. The number of non-irradiated *Puma−/−* SP cells was 1.4 fold higher than that of *WT* control and this significant difference was further increased (2.3 fold) following irradiation.

In conclusion, *Puma−/−* spermatogonia are more resistant than *WT* cells to genotoxic stress demonstrating that Puma-controlled intrinsic apoptosis has a central role. Nevertheless, the overlay of the TUNEL assay kinetics (Fig. 2a and 4a) shows that the number of dead *Trail−/−* spermatogonia was 50% higher than that of *Puma−/−* cells at 16 h. This suggests that different populations of spermatogonia can be distinguished on the basis of their death-sensitivity to DNA damage: two subsets could be eliminated *via* Puma or *via* Trail/Dr5 exclusively, and a minor subpopulation by a cooperation of both pathways.

### The extrinsic and intrinsic apoptotic pathways could be effective in different subsets of injured spermatogonia

**Figure 5:**
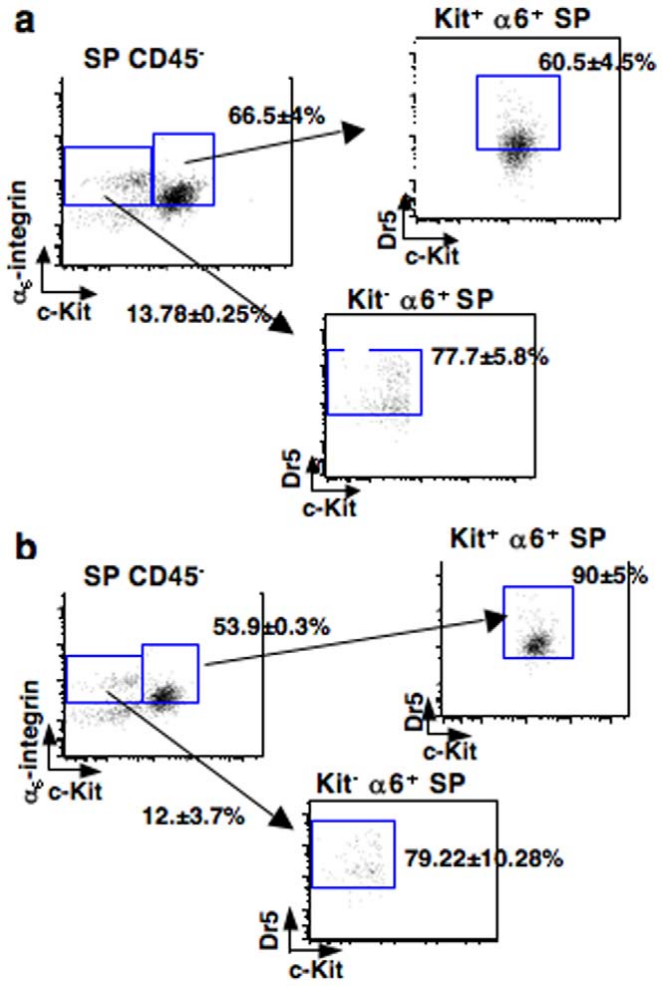
Dr5 is expressed in Kit^−^ and Kit^+^ spermatogonia and is upregulated in committed progenitors post-IR. Combined flow cytometric analysis of the expression of stem cell markers and Dr5 receptor in testicular cell suspensions. Testicular cell suspensions were stained with Ho42, and immunolabeled for the detection of α_6_-integrin, c-Kit and Dr5. Isotypic controls were performed in parallel. Hematopoietic CD45^+^ cells were excluded from the SP fraction and experiments were performed on cells (**a**) non-irradiated and (**b**) 12 h post-IR. FACS plots are gated on α_6_-integrin^+^ cells, c-Kit positive (mature progenitors) or negative (Stem cells and immature progenitors) cells and numbers indicate their percentage (±SEM).

To go further into irradiated progenitors characterization, Dr5 receptor expression was analyzed by flow cytometry on immature (Kit^–^ α_6_^+^ SP) and differentiated (Kit^+^ α_6_^+^ SP) spermatogonia, both populations expressing *Puma* RNA (data not shown). Dr5 was constitutively present on 75% of immature spermatogonia *versus* 60% of the differentiated progenitors (Fig. 5a). After irradiation, 90% of the differentiated spermatogonia were positive for Dr5 receptor, concomitant to an increase of the number of molecules per cell, as shown by fluorescence enhancement (Fig. 5b). By contrast, the number of irradiated immature cells expressing Dr5 was constant.

**Figure 6:**
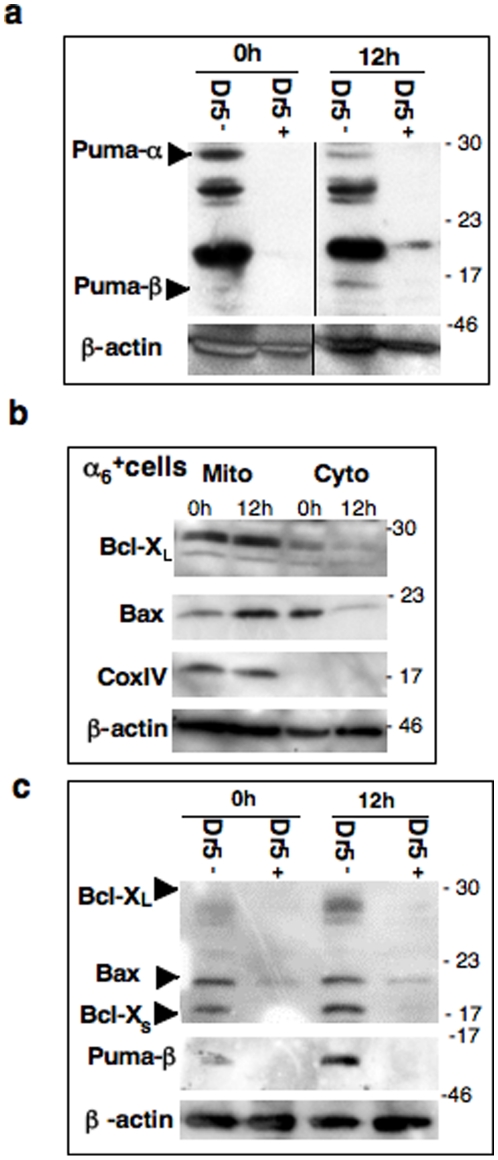
Puma is not detected in MACS Dr5-positive cells. (**a**) Differential expression of Puma isoforms in testicular Dr5^+^ and Dr5^−^ populations. Dr5 fractions were isolated by immunomagnetic purification prior and post-IR and analyzed by western-blot with β actin as loading control. Data presented are representative of 2 independent experiments and the Dr5 populations were purified from 3 males. See Figure S4, for characterization of MACS Dr5 populations. (**b**) Differential expression of Bcl-X_L_ and Bax in cytosolic and mitochondrial fractions. Testicular α_6_^+^ cells, enriched in spermatogonia after selection by immunomagnetic purification, were collected prior and 12 h post-IR and separated into cytosolic and mitochondrial-enriched fractions for western blot analysis; the sample purity was assayed using an anti-Cox IV antibody (mitochondrial marker) and β actin as loading control. Data presented are representative of 2 independent experiments and the α_6_^+^ populations were purified from 3 males. See Figure S3 for corresponding semi-quantitative RT-PCR analysis. (**c**) Bax is the only Bcl2 family member detected in both Dr5^+^ and Dr5^−^ populations.

We took advantage of the ability to separate testicular cell suspension into Dr5-positive (Dr5^+^) and Dr5-negative (Dr5^−^) fractions using Magnetic Activated Cell Sorting (MACS by Miltenyi Biotech). Spermatogonia were present in both Dr5^+^ and DR5^−^ fractions, while the former was highly more enriched (Population characterization in Figures S4 and S5). On western-blots (Fig. 6a), Puma could not be detected in Dr5^+^ fractions. By contrast, Puma-α was constitutively present in Dr5^−^ population, and appeared downregulated after irradiation. Puma-β isoform was also detected in irradiated Dr5^−^ cells within it accumulated, as previously observed in the irradiated α_6_^+^ fractions (Fig. 3b). We then focused on the Puma “partners”. Indeed, BH3-only proteins maximize their effects through oligomeric interactions with pro- and/or anti- apoptotic members of the Bcl2 family: Bcl-X_L_ and/or Bax for Puma [24]. In irradiated α_6_^+^ population (Fig. 6b), Bax was translocated to the mitochondrial-enriched extract, with a concomitant loss of its cytoplasmic form, suggesting Bax activation and apoptosis induction. By contrast, the anti-apoptotic Bcl-X_L_ was strongly expressed in the mitochondrial-enriched lysates of both control and irradiated α_6_^+^ cells. However, only Bax was detected in both Dr5^+^ and Dr5^−^ populations (Fig. 6c). Conversely, the anti-apoptotic Bcl-X_L_ accumulated in irradiated Dr5^−^ cells, while the pro-apoptotic Bcl-X_s_ appeared unaffected.

In conclusion, these data show that most of spermatogonia can be divided up into a Dr5-positive subset potentially sensitive to Trail, and another Dr5-negative subpopulation deleted *via* intrinsic pathway after DNA damage.

## Discussion

We demonstrate herein that two p53-dependent pathways are involved in radiation- induced apoptosis of spermatogonia *via* the transcriptional control of *Puma* and *Dr5* genes. The inactivation of each pathway results in partial inhibition of apoptotic death of spermatogonia, and the overlay of TUNEL-kinetics suggested the existence of several spermatogonia populations according to death sensitivities. Unfortunately we were unable to obtain double mutated animals (*Puma−/− Trail−/−*), suggesting embryonic lethality.

The Puma-controlled intrinsic pathway is induced in one subset of progenitors, independently of the Trail/Dr5 system. Puma-regulated death is widely involved after genotoxic stress: in lymphocyte lineages, small intestine and central nervous system [25]–[27]. While *Puma* transcription levels were highly elevated in spermatogonia, Puma protein amounts remain limiting: (i) irradiated *Puma+/−* spermatogonia demonstrate an intermediate resistance to death, as previously reported for somatic cells [28], (ii) no obvious variation of Puma-α quantity is detected whatever the p53 status and this isoform is mainly located in the mitochondrial –enriched fraction of α_6_^+^ cells, treated or not. Our results suggest that the mitochondrial apoptosis could be induced by Puma-β that only accumulates in *WT* cells, illustrating the p53 dependence. Alternative splicing of *Puma* is conserved among species, but the preference for the isoform β could be male germ cell-specific. Indeed, in cisplatin-treated kidney cells, only isoform α is upregulated according to p53 status [29]. Puma-β markedly differs at the N-terminus from Puma-α [30], however except its mitochondrial location little is known about its activity (partners, apoptotic role…). Depending on the experimental systems, two different models for Puma action have been proposed: (i) a displacement of Bcl-X_L_ to release constitutively active Bax, or (ii) a direct interaction with Bax to induce its activation [22], [30]. These potent partners are detected in α_6_^+^ cells: the pro-survival Bcl-X_L_ was mainly located at mitochondria and the pro-apoptotic Bax translocates to mitochondrial membrane suggesting the conformational activation necessary to apoptosis induction. But they are differently distributed between Dr5-negative and Dr5-positive populations; the later undergoes extrinsic death via Trail/Dr5 without expression of Puma isoforms.

As for somatic cells, DNA damage upregulates Dr5 expression on spermatogonia in a p53-dependent manner, and injured adult testicular *Trail−/−* progenitors are resistant to apoptosis, like somatic *Dr5−/−* tissues [9]. The presence of *Trail* RNA in both Sertoli and α_6_^+^SP cells suggests that spermatogonia can undergo apoptosis in an autocrine and/or paracrine manner. The Trail/Dr5 pathway can be associated with induction of procaspase-8 cleavage in α_6_^+^ cells (Figure S1). But the presence of Bax in Dr5^+^ population, as well as Bid truncation in irradiated α_6_^+^ cells (Data not shown), support that mitochondria might be recruited to amplify the process in normal testicular cells, as in cancer lines [31]. Nevertheless, our data do not allow the exclusion of a minor third sub-population exhibiting both death pathways.

These distinct populations of spermatogonia exist according to “death criteria” and appear to overlap with the classical distribution of spermatogonia. Indeed, among immature spermatogonia (Kit^−^ α_6_^+^SP), 75% express Dr5 receptor while Puma protein was not detected in the MACS-Dr5^+^ population. Irradiation does not modify Puma specific expression. We cannot exclude that part of the 25% Dr5-negative immature cells could be Puma-positive and/or Bcl-X_L_ positive. It is important to note that undifferentiated spermatogonia include germinal stem cells (As) that resist to radiation-induced death and are responsible for differentiation recovery. Consequently, either they express Dr5 receptor and the signaling pathway might be inhibited after moderate stress, or they are negative for Dr5 and Bcl-X_L_ accumulation could be a way to resist to death induction. The lack of suitable anti-Puma antibody for *in situ* studies did not allow co-labeling with stem cell marker (e.g. Plzf [15]) to refine characterization.

Conversely, the differentiated spermatogonia (Kit^+^ α_6_^+^SP) contain about 60% of Dr5^+^ cells, with a 30% increase post IR. This enhancement in Dr5^+^ progenitors could be due to a real Dr5 upregulation, but also to the rapid death of Dr5^−^ differentiated progenitors, potentially expressing Puma, thereby causing an apparent enrichment in more radio-resistant Dr5^+^ cells at 12 h post-IR.

These data raise few questions: (i) the constitutive presence of Dr5 on most of the immature spermatogonia suggests a role of Trail/Dr5 in differentiation entry; this is not surprising as this pathway has been reported to have regulatory functions in the immune and hematopoietic compartments, as well as in bone remodeling and muscle differentiation [32]–[35]. (ii) Spermatogonia death could be regulated according to a threshold mechanism: the intrinsic pathway would be activated rapidly and/or by low damage, and the extrinsic pathway could be recruited over a major stress level. It is important to note that the applied γ dose (1.6 Gy) induces a transient sterility. In conclusion, adult spermatogonia exhibit different sensitivities to DNA damage linked to specific metabolisms of reactive oxygen species, to DNA repair capacities and/or to cycling status. The less mature spermatogonia are more resistant to radiation-induced stress than committed progenitors, but over a critical damage threshold the extrinsic pathway could be activated, thereby limiting the mutational risk.

## Materials and Methods

### Mice

The *Fas*−/− [36], *Trail−/−* [37] and *Puma−/−* [38] mouse strains were raised on a C57BL/6 background in our animal facilities, as well as the *p53*−/− [39] strain on SV129 background. Whatever their genotypes, all the KO males are fertile. Since adult C57BL/6 germ cells are more sensitive to radiation-induced death than the SV129 cells, control and mutants of same genetic background were used in experiments. Adult males (2.5- to 3.5- months-old) were exposed to 1.5 or 2 Gy γ rays in a ^60^Co irradiator (dose rate 0.085Gy/mn). The Figure S1 describes the evolution of some death markers in irradiated SV129 testis. All animal procedures were carried out in accordance with French Government policies (Services Vétérinaires de la Santé et de la Production Animale, Ministère de l′Agriculture; N° A92-032-02).

### Sertoli cell-enriched cultures

Primary cultures enriched in adult Sertoli cells were prepared by trypsin/collagenase I digestion of testes from 3- week-old males [40]. Isolated cells were seeded in DMEM-F12 medium supplemented with 15 mM Hepes pH 7.2, 1.2 g/L Na_2_CO_3_, 5 ng/mL EGF, 5 µg/mL transferin and 10 µg/mL bovine insulin, at a density of 5×10^5^ cells/cm^2^ and maintained at 32°C for 48 h. A hypotonic shock in 20 mM Tris-HCl pH 7.4 (2.5 min at RT) allowed removal of most of germ cells. After one HBSS rinse, the adherent Sertoli cells were recovered for 48 h in the enriched DMEM-F12 medium and assayed by RT-PCR amplification for the expression of the germ cell marker *c-Kit*.

### Testicular single-cell suspensions

Germ cells are prepared as previously described [18]. Briefly seminiferous tubules are dispersed in collagenase I at 32°C. Interstitial cells were further discarded by filtration, and the seminiferous tubules are digested in Cell dissociation buffer (Invitrogen). After washing and filtration, isolated cells are resuspended in (HBSS supplemented with 20 mM Hepes pH 7.2, 1.2 mM MgSO_4_7H_2_0, 1.3 mM CaCl_2_2H_2_0, 6.6 mM sodium pyruvate, 0.05% lactate, glutamine and 1% FCS) and incubated at 32°C.

### Real-Time PCR amplification from sorted cells

The testicular cell suspension is enriched in α_6_-integrin positive cells using immunomagnetic purification (MACS by Miltenyi Biotech) [18]. The α_6_^+^ cellular fraction is incubated (50 min. at 32°C) with Ho42 and further labeled for CD45 (clone 30-F11, Becton Dickinson; 15 min at 4°C). The hematopoietic CD45-PE^+^ cells were discarded during FACS analysis and α_6_^+^SP population was sorted with a FACStar Plus Flow Cytometer (Becton Dickinson). Populations were independently purified from 5 *WT* and 3 *p53−/−* mice and RNA further stoechiometrically pooled. RNA purification, and cDNA synthesis were performed as previously described [18], [21].

For cDNA synthesis the following primers were used:

*Dr5* (CTgTgCATTCgTCTCTCTTgg/TgAgTCgTTTCCgTTTACCg); *Trail* (gTgTCTgTggCTgTgACTTACA/AATgCCCTTTCCgAgAggA); *Puma* (CACCCCATCgCCTCCTTTCT/ggAAggggCgCggACTgTCg); *Noxa* (TggAgTgCACCggACATAACT/CACTCgTCCTTCAAgTCTgCTg); *Bax* (TCAAggCCCTgTgCACTAA/TgAggACTCCAgCCACAAA); *Bcl-X_L_* (gATgCAggTATTggTgAgTCgg/ATCCACAAAAgTgTCCCAgCC); *Bid* (CTTTgCTCCgTgATgTCTTCC/TgCgggCTCCTCAgTCCATC); *Gusβ* (AgCCACAgCTgAATAgCCAgTT/ACTCCTCACTgAACATgCgAgg); *βActin* (TCgTgCgTgACATCAAAgAgA/gAACCgCTCgTTgCCAATAgT).

Real-time PCR amplifications were carried out with the Sybr Green® PCR kit (Applied Biosystems) and each reaction was performed at least twice in duplicate using a Abiprism 7000 apparatus. The *β glucuronidase* (*Gusβ*) gene was used as an internal control because *Gusβ* levels were constant in *WT* α_6_^+^SP fractions whatever the p53 status. A second internal control, *βactin* (Mm00446953m1), was used in Taqman® assays (Applied Biosystems) to compare *WT* and *p53−/−* samples. After normalization, mean expressions were shown as fold increase (± SEM), after comparison with the mean expression levels of the *WT* control to which was ascribed an arbitrary value of 1.

### Immuno-labeling and absolute SP cell count

For marker analysis one million testicular cells were firstly stained with Ho42 in 1 mL incubation buffer for 45 min. at 32°C, and then labeled with 1 µg of antibody (PE- anti-DR5 (MD5-1, eBioscience), or FITC-anti-human-CD49f, APC-anti-Kit and Pc5-anti-CD45 (BD Pharmingen)) for 15 min at 4°C. Isotype antibodies were used as negative controls and propidium iodide (PI) was added prior to analyses with a LSRII cytometer (Becton Dickinson). Mean percentages are shown (± SEM).

For population measurements, after Ho42 and PI staining of testicular cell suspensions, SP fractions were quantified by FACS using fluorescent TruCOUNT™ beads (BD Biosciences). A minimum of three different counts was performed per testis prior to and 24 h post-IR in the presence of the fluorescent beads as an internal reference. Elongated spermatids were excluded during the analysis to avoid any contamination of the SP.

### TUNEL assay

TUNEL assay was performed as previously described [3] and labelings were repeated 3 times, with at least 200 tubules were counted per male. Cells located at the basal membrane were counted under an Olympus BX51 photomicroscope equipped with an Insight QE Spot cameram.

### Protein purification and western blotting

The testicular cell suspensions are enriched in either in α_6_-integrin positive cells (anti-CD49- PE), or in Dr5 positive cells (anti-Dr5 PE) using immunomagnetic purification (MACS by Miltenyi Biotech). For total protein lysates, MACS purified fractions were lysed for 30 min at 4°C in 25 mM Tris pH 7.5, 2 mM EDTA, NP40 5% (v/v), 0.6 M NaCl, 15 mM NaF, Na_3_VO_4_ with protease inhibitor cocktail (Roche Diagnostics) then centrifuged for 30 min at 16 000 g, 4°C. In order to purify mitochondrial proteins from α_6_^+^ populations, the ProteoExtract® Cytosol/mitochondria kit (Calbiochem) was used with a 40 times dounce according to the manufacturer's instructions. Concentrations were measured with the Quant-it™ Protein assay kit `(Invitrogen). After migration of 30 µg of proteins on 12 or 14% SDS-Page gels, they were blotted on Immobilon™P^SQ^ membranes using 35% methanol solution at the anode. Membranes were hybridized with anti-bactin (AC15, Sigma-Aldrich), anti-CoxIV (20E8, Molecular Probes), anti- Bcl-X_L_ (S-18), anti-Bax (N-20) (Santa Cruz), anti-Puma (rabbit polyclonal 9643 (Abcam) or 4976 (Cell Signaling)) in TBS-milk. Proteins were visualized with peroxidase-coupled secondary antibodies (Pearce) using the ECL Plus System (GEHealth) on Kodak films. Puma-α isoform was identified with the 9643 antibody, using *Puma−/−* cell lysate as a negative control [41] and Puma-β isoform was detected with the 4976 antibody. β-actin was used as loading control and sample purity was assayed using an anti-Cox IV antibody (mitochondrial marker). Data presented are representative of 2 independent experiments and MACS-α_6_^+^ population and MACS-Dr5 populations were purified from 3 males. Characterization of MACS-Dr5 cell populations is presented in Figure S4.

### Statistical analysis

Values were expressed as the mean ± standard error (±SEM). Unpaired Student's *t* test was used to compare data (Kaleidagraph, Synergy software). Taking into account the variance distribution, a p<0.05 was considered significant.

## Supporting Information

Figure S1 Detection of apoptotic markers in irradiated testes. On the basis of TUNEL dose-response previously described by Hasegawa M, Wilson G, Russell LD, Meistrich ML (1997) Radiation-induced cell death in the mouse testis: relationship to apoptosis. Radiation research. 147: 457–467. Immunohistochemistry on SV129 testes, Immunostaining was performed on 5microm sections fixed in Bouin. After antigen retrieval by microwave irradiation in citrate buffer, sections were treated with 0.3% H2O2 in PBS and blocked with 3% BSA in PBS. They were then incubated with anti-cleaved caspase-9 (Asp353), -cleaved caspase-7 (Asp198) (Cell Signaling), -cytochrome c (6H2B4, Becton Dickinson) or -p53 (CM5, Novocastra) antibodies diluted in BSA overnight at 4°C. Labeling was revealed using the ABC Vectastain Kit (Vector Laboratories) and Sigma fast DAB (Sigma). Sections were counterstained with hematoxylin and analyzed under an Olympus BX51 photomicroscope equipped with an Insight QE Spot camera. (A) P53 is stabilized 4 h post-IR in spermatogonia, (B) Cytochrome c is released 7 h post-IR in some spermatogonia. Arrows indicate positive spermatogonia (bar = 20 microm). (C) Detection of dead spermatogonia 16h-post IR by TUNEL assay. (D) Cleavage of pro-caspase-9 and -7 in spermatogonia, with antibodies specifically recognizing the processed form. Arrows indicate positive spermatogonia (bar = 20 microm). (E) Processing of pro-caspase-2 and -8 followed by immunoblotting of whole lysates of MACS- alpha6+ fraction (enriched in spermatogonia) versus MACS - alpha6- fraction (including spermatocytes I, spermatocytes II and spermatids) prior to and post-IR; actin was used as a loading control. Representative results obtained from 3 male pooled testicular cells at each time point. Each experiment was performed at least twice. Membranes were hybridized with anti-caspase- 8 (1G12) and anti-caspase- 2 (11B4) (Alexis), anti-betaactin (AC15, Sigma-Aldrich) and proteins were visualized with peroxidase-coupled secondary antibodies (Pearce) using the ECL Plus System (GEHealth) on Kodak films.
(0.96 MB TIF)

Figure S2 Flow cytometric analysis of Dr5 labeling on testicular cell suspension. (a) Testicular suspension is analyzed according to Ho42 and PI staining; viable cells are gated in R1, and dead cells are excluded. (b) Elongated spermatids are excluded from the viable testicular cells (gate R2). (c) The SP cells are gated, and further analyzed for Dr5 labeling (d) according to the isotypic threshold (gate R4).
(0.14 MB TIF)

Figure S3 Analysis of the expression levels of Noxa, Bid, Bcl-XL and Bax genes. Comparison in p53+/+ or p53−/− sorted CD45- alpha6+ SP cells, by semi-quantitative RT-PCR prior to, 7 and 12 hours after irradiation. Each expression level was normalized to its value in non-irradiated cells and is indicated as a fold increase (± SEM, 3 separate experiments from pooled CD45- alpha6+ SP populations, independently purified from 5 males). On left panel, expression of the anti-apoptotic Bcl-XL gene did not vary significantly, like RNA levels of the pro-apoptotic BH3-only members Bid and Noxa, potently involved in DNA damage response. On right panel, expression of the pro-apoptotic Bax gene was significantly reduced in irradiated CD45- alpha6+ SP cells (black star p = 0.00031). The basal level in p53−/− cells was significantly increased (p = 0.0028).
(0.09 MB TIF)

Figure S4 Characterization of Dr5+ and Dr5- cell populations Total testicular sample was purified by MACS according to Dr5 expression. MACS cellular fractions were cytospined, fixed with 2% PFA-PBS and stained with anti-CD9, anti-Kit (Becton Dickinson) or anti-Plzf after Triton permeabilization (Santa Cruz). After incubation with a secondary antibody coupled to Alexa 488, cells were counterstained with Dapi. Kit is a marker of differentiated spermatogonia, and not of testicular stem cells. CD9 is expressed on spermatogonia and also on stem cells, which are specifically expressing plzf. Most of the Kit- spermatogonia are Plzf+.
(0.89 MB TIF)

Figure S5 (0.84 MB TIF)
