# Supplementary figures and images for: Puma and Trail/Dr5 Pathways Control Radiation-Induced Apoptosis in Distinct Populations of Testicular Progenitors
Source: PLoS One. 2010 Aug 12;5(8):e12134. doi: 10.1371/journal.pone.0012134 (PMC2920820; doi:10.1371/journal.pone.0012134)

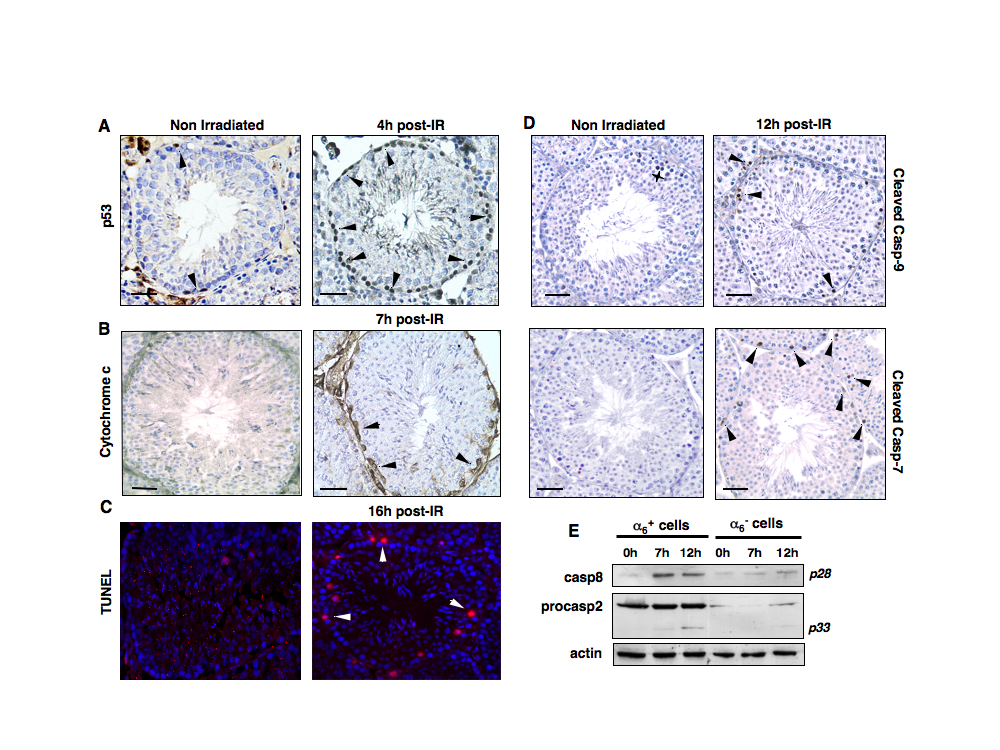

Supplement: Figure S1 — Detection of apoptotic markers in irradiated testes. On the basis of TUNEL dose-response previously described by Hasegawa M, Wilson G, Russell LD, Meistrich ML (1997) Radiation-induced cell death in the mouse testis: relationship to apoptosis. Radiation research. 147: 457–467. Immunohistochemistry on SV129 testes, Immunostaining was performed on 5microm sections fixed in Bouin. After antigen retrieval by microwave irradiation in citrate buffer, sections were treated with 0.3% H2O2 in PBS and blocked with 3% BSA in PBS. They were then incubated with anti-cleaved caspase-9 (Asp353), -cleaved caspase-7 (Asp198) (Cell Signaling), -cytochrome c (6H2B4, Becton Dickinson) or -p53 (CM5, Novocastra) antibodies diluted in BSA overnight at 4°C. Labeling was revealed using the ABC Vectastain Kit (Vector Laboratories) and Sigma fast DAB (Sigma). Sections were counterstained with hematoxylin and analyzed under an Olympus BX51 photomicroscope equipped with an Insight QE Spot camera. (A) P53 is stabilized 4 h post-IR in spermatogonia, (B) Cytochrome c is released 7 h post-IR in some spermatogonia. Arrows indicate positive spermatogonia (bar = 20 microm). (C) Detection of dead spermatogonia 16h-post IR by TUNEL assay. (D) Cleavage of pro-caspase-9 and -7 in spermatogonia, with antibodies specifically recognizing the processed form. Arrows indicate positive spermatogonia (bar = 20 microm). (E) Processing of pro-caspase-2 and -8 followed by immunoblotting of whole lysates of MACS- alpha6+ fraction (enriched in spermatogonia) versus MACS - alpha6- fraction (including spermatocytes I, spermatocytes II and spermatids) prior to and post-IR; actin was used as a loading control. Representative results obtained from 3 male pooled testicular cells at each time point. Each experiment was performed at least twice. Membranes were hybridized with anti-caspase- 8 (1G12) and anti-caspase- 2 (11B4) (Alexis), anti-betaactin (AC15, Sigma-Aldrich) and proteins were visualized with peroxidase-coupled se [file pone.0012134.s001.tif]

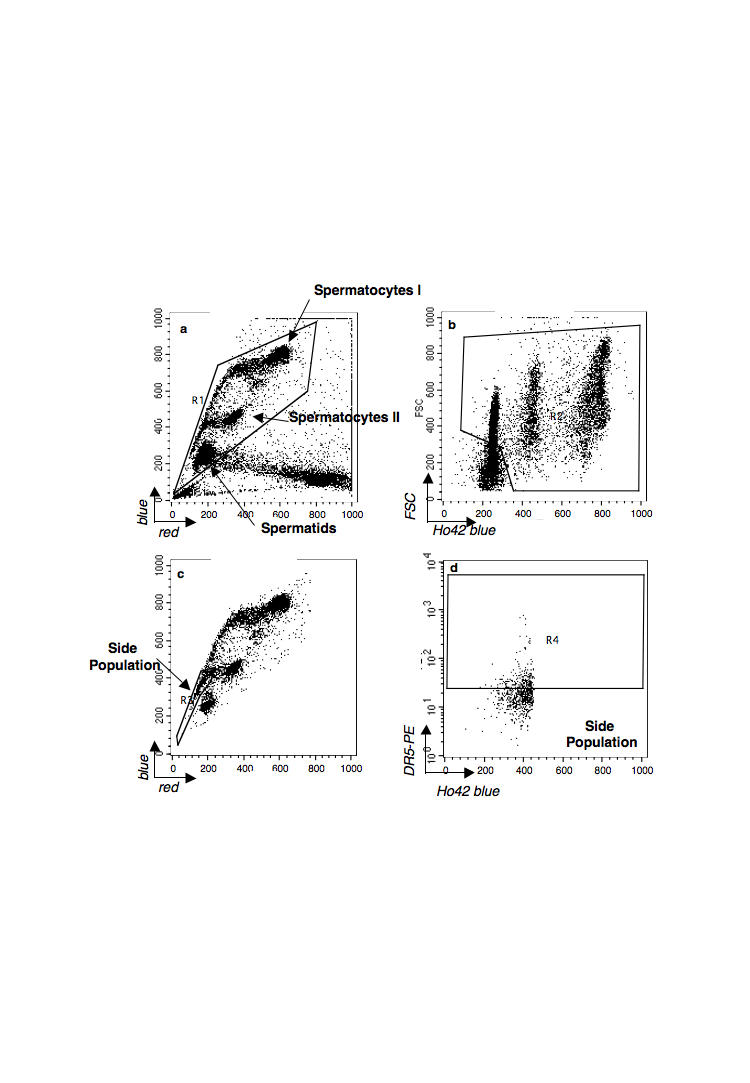

Supplement: Figure S2 — Flow cytometric analysis of Dr5 labeling on testicular cell suspension. (a) Testicular suspension is analyzed according to Ho42 and PI staining; viable cells are gated in R1, and dead cells are excluded. (b) Elongated spermatids are excluded from the viable testicular cells (gate R2). (c) The SP cells are gated, and further analyzed for Dr5 labeling (d) according to the isotypic threshold (gate R4). (0.14 MB TIF) [file pone.0012134.s002.tif]

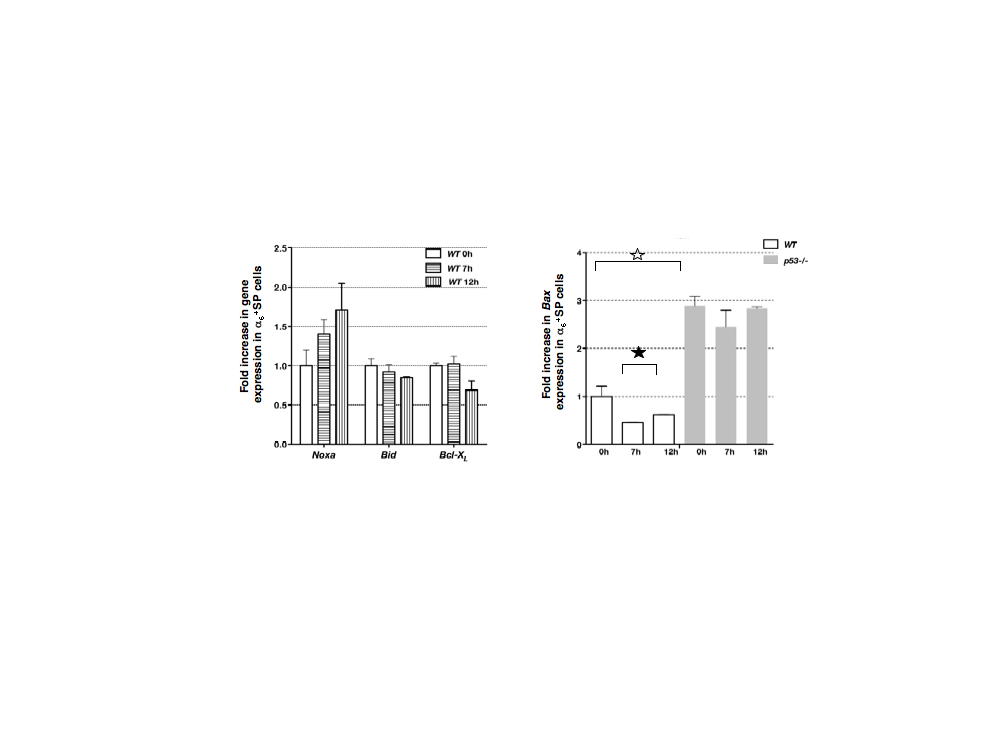

Supplement: Figure S3 — Analysis of the expression levels of Noxa, Bid, Bcl-XL and Bax genes. Comparison in p53+/+ or p53−/− sorted CD45- alpha6+ SP cells, by semi-quantitative RT-PCR prior to, 7 and 12 hours after irradiation. Each expression level was normalized to its value in non-irradiated cells and is indicated as a fold increase (± SEM, 3 separate experiments from pooled CD45- alpha6+ SP populations, independently purified from 5 males). On left panel, expression of the anti-apoptotic Bcl-XL gene did not vary significantly, like RNA levels of the pro-apoptotic BH3-only members Bid and Noxa, potently involved in DNA damage response. On right panel, expression of the pro-apoptotic Bax gene was significantly reduced in irradiated CD45- alpha6+ SP cells (black star p = 0.00031). The basal level in p53−/− cells was significantly increased (p = 0.0028). (0.09 MB TIF) [file pone.0012134.s003.tif]

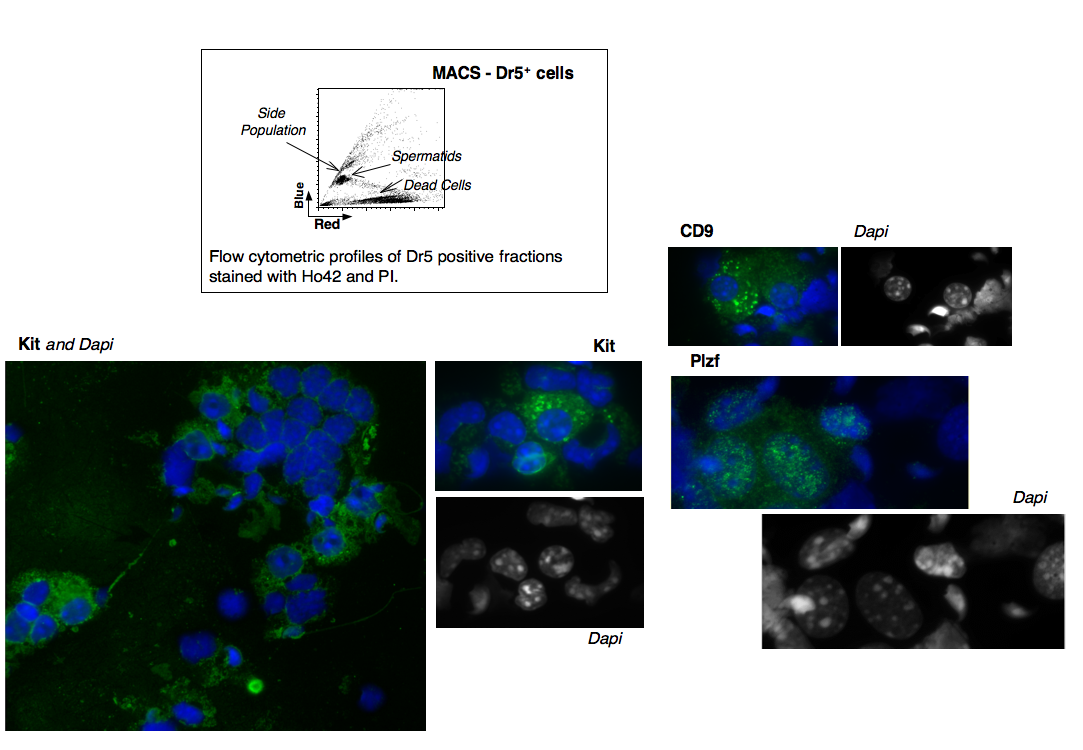

Supplement: Figure S4 — Characterization of Dr5+ and Dr5- cell populations Total testicular sample was purified by MACS according to Dr5 expression. MACS cellular fractions were cytospined, fixed with 2% PFA-PBS and stained with anti-CD9, anti-Kit (Becton Dickinson) or anti-Plzf after Triton permeabilization (Santa Cruz). After incubation with a secondary antibody coupled to Alexa 488, cells were counterstained with Dapi. Kit is a marker of differentiated spermatogonia, and not of testicular stem cells. CD9 is expressed on spermatogonia and also on stem cells, which are specifically expressing plzf. Most of the Kit- spermatogonia are Plzf+. (0.89 MB TIF) [file pone.0012134.s004.tif]

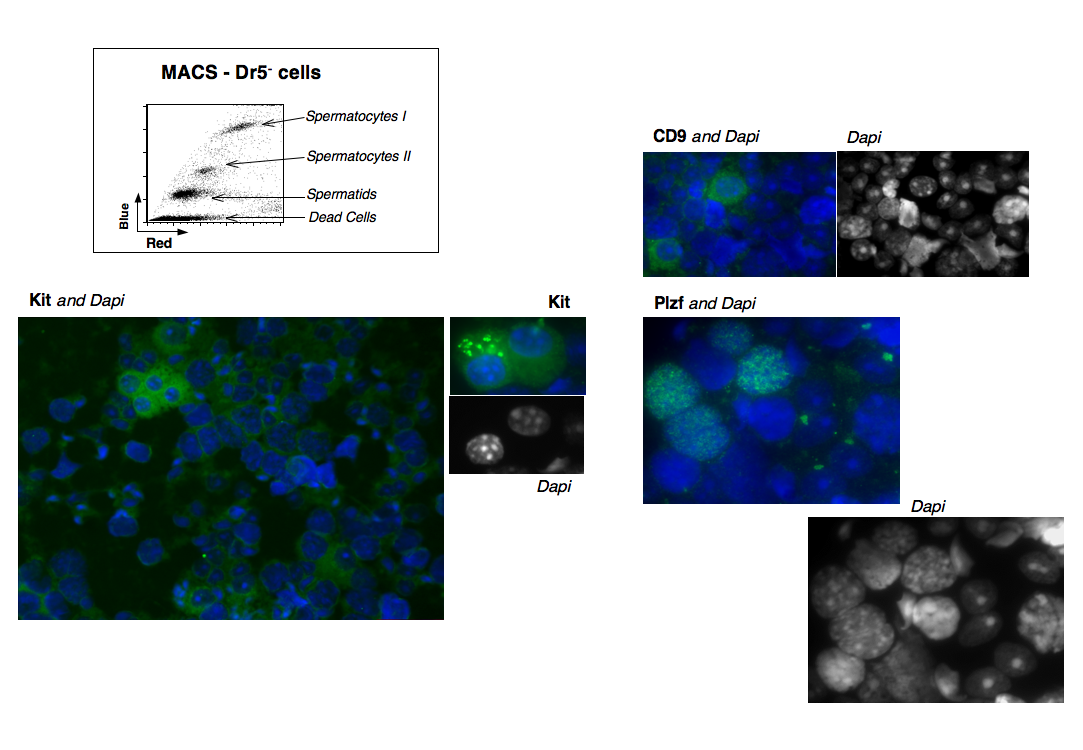

Supplement: Figure S5 — (0.84 MB TIF) [file pone.0012134.s005.tif]
